# The speciation of copper (II)/ethylenediamine/oxalate system by evolving factor analysis

**DOI:** 10.1155/S1463924695000265

**Published:** 1995

**Authors:** Shouxin Ren, Ling Gao

**Affiliations:** Department of Chemistry Inner Mongolian University Inner Mongolia Huhehot 010021 China

## Abstract

Principal component analysis and evolving factor analysis were applied to the study of the speciation of Cu (II)/ethylenediamine/ oxalate. Two programs were designed, based on mathematical algorithms. Error functions were calculated for evaluating the number of species. Submatrix analysis plots were constructed to estimate the species present in the system. The method should prove useful in studies of complex systems in environmental samples.


Journal of Automatic Chemistry, Vol. 17, No. 5 (September-October 1995), pp. 173-177

### The speciation of copper (II)/ethylenediamine/oxalate system by evolving factor analysis

**Shouxin Ren and Ling Gao**

*Department of Chemistry, Inner Mongolian University, Huhehot 010021, Inner Mongolia, China*

*Principal component analysis and evolving factor analysis were applied to the study of the speciation of Cu (II)/ethylenediamine/ oxalate. Two programs were designed, based on mathematical algorithms. Error functions were calculated for evaluating the number of species. Submatrix analysis plots were constructed to estimate the species present in the system. The method should prove useful in studies of complex systems in environmental samples.*

### Introduction

Information about the speciation of metals in natural water is important in studies of the toxicity of metals for aquatic organisms. The study of speciation also contributes to the understanding of trace metal transport in the water environment. Biological effects on aquatic organisms and geochemical behaviour both depend greatly on the species of the element so the study of speciation has been one of the most important areas of environmental and analytical chemistry in recent years.

Studies of multiple equilibria in solution have solved a number of problems in speciation. Traditional approaches use least squares methods based on a chemical model and on compliance with the law of mass action [1]. A variety of instrumental techniques have been used for the determination of the number, nature and stabilities of multiple equilibria systems. Spectrophotometry has considerable advantages over the other techniques because of its simplicity. Gampp *et al.* [2] proposed a model-free method derived from principal component analysis (PCA): evolving factor analysis (EFA). EFA is a powerful method for analysing multivariate data with an intrinsic order produced by many modern hyphenated instruments, such as speciation in multiple equilibria systems using spectrophotometric titration. Data can be listed, according to its pH, from low to high. The first columns may contain the data measured at low pH values and the last columns those obtained at high pH values. The method is based upon repetitive eigenvalue analysis of a set of data matrices obtained during the evolutionary process. Eigenanalysis is performed on a complete series of matrices, which are constructed by successively adding spectra to the previous matrix during the evolutionary process. As new absorbing species evolve, the eigenvalues of the abstract factors increase by an order of magnitude. The logarithmic eigenvalues are plotted as a function of the progessing titration. Every submatrix is calculated by PCA in turn.

### Experimental

### *Instruments and apparatus*

The Shimadzu UV-265 and UV-120 spectrophotometers were used for all experiments; a GW 286 EX/16 microcomputer with a maths coprocessor was used for the calculations; and a Mettler DL21 titrator was used for standardization of standard solutions.

### *Reagents*

All reagents were of analytical reagent grade. Doubly distilled and de-ionized water were used. Standard solutions of copper(II) nitrate, ethylenediamine and potassium oxalate were prepared and standardized according to generally accepted procedures.

### *Spectrophotometric titration*

Solutions which contain Cu(II) ion and one or two of the ligands were titrated with a base at a constant ionic strength (0·5M) and temperature (25°C). After each addition of titrant and equilibration, the pH was measured. The absorbance of spectra of each solution was measured at a different pH, with a wavelength range from 480 nm to 820 nm, in 20 nm intervals. An experimental data matrix, $D$, was built up from these data.

### *Computer programs and their algorithm*

The steps of the algorithm were as follows:

(1) *Building the data matrix* $D$ $(N, M)$. Matrix $D$ contains the spectra at $N$ wavelengths of the $M$ mixtures obtained at the successive titration points of the spectrometric titration.

(2) *Factor analysis of the experimental data matrix* $D$. The variance-covariance matrix $A(D^TD)$ can be subjected to single value decomposition for calculation of the eigenvalues and eigenvectors. The number of species is estimated by several criteria based on the theory of error in factor analysis [3].

(3) *Evolving factor analysis.* The fundamental idea of EFA is to follow the evolution of the rank of the data matrix $D$ as a function of the ordered variable, for example pH in a spectrophotometric titration. This is done by PCA on an increasing data matrix. The logarithm of the singular values corresponding to the principal components determined by PCA versus increasing and decreasing pH, are plotted in the same graph.

0142-0453/95 $10.00 © 1995 Taylor & Francis Ltd.

*Table 1. Summary of the conditions for the experimental determinations.*

| System | Molar concentrations | | | Wavelength range (nm) | pH range |
|---|---|---|---|---|---|
| | Cu(II) | ox | en | | |
| Cu(II)/ox | 0·0100 | 0·1000 | — | 480–820 | 3·5–10·0 |
| Cu(II)/en | 0·0100 | — | 0·1000 | 480–820 | 4·0–11·0 |
| Cu(II)/ox/en | 0·0100 | 0·1000 | 0·1000 | 480–820 | 3·5–11·0 |

*Table 2. Principal component analysis applied to Cu(II)/ox/en system.*

| n | EV | RE | IE | XE | IND | ER | Frac | REV | REV ratio |
|---|---|---|---|---|---|---|---|---|---|
| | × 10³ | | | | | | | | |
| 1 | 6·3025 | 0·0675 | 0·0169 | 0·0654 | 0·3002 | 5·5629 | 0·844 19 | 0·023 170 93 | 4·92 |
| 2 | 1·1330 | 0·0113 | 0·0040 | 0·0106 | 0·0576 | 38.4695 | 0·151 75 | 0·004 720 65 | 33·66 |
| 3 | 0·0295 | 0·0020 | 0·0009 | 0·0028 | 0·0116 | 83·8337 | 0·003 95 | 0·000 140 24 | 72·66 |
| 4 | 0·0004 | 0·0016 | 0·0008 | 0·0014 | 0·0109 | 2·0273 | 0·000 05 | 0·000 001 93 | 1·74 |
| 5 | 0·0002 | 0·0013 | 0·0007 | 0·0011 | 0·0109 | 1·4798 | 0·000 02 | 0·000 001 11 | 1·25 |
| 6 | 0·0001 | 0·0011 | 0·0007 | 0·0009 | 0·0111 | 1·7345 | 0·000 02 | 0·000 000 88 | 1·45 |
| 7 | 0·0001 | 0·0010 | 0·0006 | 0·0007 | 0·0119 | 1·4184 | 0·000 00 | 0·000 000 61 | 1·16 |
| 8 | 0·0000 | 0·0008 | 0·0006 | 0·0006 | 0·0131 | 1·4847 | 0·000 00 | 0·000 000 52 | 1·19 |
| 9 | 0·0000 | 0·0007 | 0·0005 | 0·0005 | 0·0148 | 1·2979 | 0·000 00 | 0·000 000 45 | 1·01 |
| 10 | 0·0000 | 0·0006 | 0·0005 | 0·0004 | 0·0170 | 1·8138 | 0·000 00 | 0·000 000 44 | 1·36 |
| 11 | 0·0000 | 0·0005 | 0·0004 | 0·0003 | 0·0215 | 1·2982 | 0·000 00 | 0·000 000 32 | 0·93 |
| 12 | 0·0000 | 0·0005 | 0·0004 | 0·0002 | 0·0284 | 1·3170 | 0·000 00 | 0·000 000 35 | 0·88 |
| 13 | 0·0000 | 0·0003 | 0·0003 | 0·0001 | 0·0384 | 1·9767 | 0·000 00 | 0·000 000 40 | 1·19 |
| 14 | 0·0000 | 0·0002 | 0·0002 | 0·0001 | 0·0614 | 2·0204 | 0·000 00 | 0·000 000 34 | 1·01 |
| 15 | 0·0000 | 0·0001 | 0·0001 | 0·0000 | 0·0582 | 34·6303 | 0·000 00 | 0·000 000 34 | 11·54 |
| 16 | 0·0000 | | | | | | 0·000 00 | 0·000 000 02 | |

The plots perform the submatrix analysis of the titration data. Two programs, SPGRAFA and SPGREFA, which were based on the algorithm, were designed to perform the calculation.

### Results and discussion

Three different systems were used in this work:

(1) Cu(II)/oxalate (ox).
(2) Cu(II)/ethylenediamine (en).
(3) Cu(II)/ox/en.

The initial conditions used in the spectrophotometric titrations are shown in table 1. High ratios of ligand to copper ion were used in order to avoid copper hydroxide precipitation at neutral pH.

### *Estimating the number of species by factor analysis*

Nine criteria were used to estimate the number of species. Table 2 shows the principal component analysis applied to the Cu(II)/ox/en. When six components were considered, the real error or residual standard deviation function, RE, had a value of 0·0011; the imbedded error function, IE, had a value of 0·007; and the extracted error, XE, had a value of 0·0009. In general, the RE function with values larger than 0.001 is for the expected number of components, thus a range somewhat above this value is the boundary between wanted and unwanted information. Malinowski's empirical factor function, IND, reached a minimum betweeen 4 and 6; the function increased rapidly after $N = 6$. A maximum of the eigenvalue ratio function, ER, appeared at 6 [4]. In 1987, Malinowski proposed calculation of reduced eigenvalues, REV [5]; REV values are calculated according to the equation:

$$\mathrm{REV}_j = \lambda_j/(l - j + 1)(k - j + 1)$$

where $l$ and $k$ are the number of rows and columns in matrix $A$, assuming that $l$ is greater than $k$; $\mathrm{REV}_j$ is the $j$th reduced eigenvalue; and $j$ is the order number. The magnitude of REV decreased rapidly in the system until stabilizing at $N = 6$. As recommended by Gemperline and Hamilton [6], the REV ratio can be used to estimate the number of significant components in data matrix. Starting with the least significant eigenvalues, $j = k$, $\mathrm{REV}_j$ is calculated. The ratio $Y_{j-1}$ is obtained according to the following equation:

$$Y_{j-1} = \mathrm{REV}_{j-1}/\mathrm{REV}_j$$

When $\lambda_j$ and $\lambda_{j-1}$ are error eigenvalues, then the ratio $Y_{j-1}$ is approximately equal to one. If the REV ratios from $Y_5$ to $Y_{14}$ are approximately equal to one, then the eigenvalues from $N = 5$ are all the error eigenvalues. The newer indicator function $\mathrm{Frac}_i$ suggested by Malinowski is given by the equation:

$$\mathrm{Frac}_i = \lambda_i \Big/ \sum_{j=1}^{k} \lambda_j$$

where $\lambda_i$ is the $i$th eigenvalue, and the sum is over that eigenvalue and all the remaining eigenvalues. The appropriate number of components is one less than that giving the minimum $\mathrm{Frac}_i$ value. In the system, the Frac had a minimum at $\mathcal{N} = 7$, after 7 the change in Frac was quite small, suggesting that 6 was the optimum component number. From these criteria, it was concluded that six absorbing species were present.

SPGRAFA was also used to deduce the number of components for Cu(II)/en and Cu(II)/ox systems. For the Cu(II)/en system, the results indicated that no appreciable decreases in RE, IE and XE values were observed from the number of components $\mathcal{N} = 3$ to 14. A maximum of the ER function appeared at $\mathcal{N} = 3$. Although the IND function was not at a minimum at $\mathcal{N} = 3$, at $\mathcal{N} = 4$ the largest difference for this function appeared between $\mathcal{N} = 2$ and 3, which indicated that three absorbing components were present. The magnitude of the first three eigenvalues were larger that those of $\mathcal{N} = 4$–14. The magnitude of the eigenvalue represents the importance of the contribution that each eigenvector makes to fitting the real data. Therefore, three species were formed in the system. Chemical reasoning may deduce the formation of Cu(II), $[Cu(en)]^{2+}$ and $[Cu(en)_2]^{2+}$. Similar results were obtained for the Cu(II)/ox system with SPGRAFA. Three absorbing species were present in the mixture solution; these could have been Cu(II), [Cu(ox)] and $[Cu(ox)_2]^{2+}$. There is an inherent difficulty in trying to determine the exact number of species in complicated systems simply by observing the error functions; submatrix analysis plots at varying pHs are more useful for this.

### *Evolving factor analysis of Cu(II)/oxalate/ethylenediamine system*

As the first step, PCA is performed on the submatrix $D_i$ to calculate the singular value (SV) of the first $i = 1, 2$–16. The SVs obtained from backward EFA on the last $i = 1, 2$–16 spectra were calculated in a similar way.

It was assumed that the least SVs were zero and the results of the forward and backward EFAs are given in tables 3 and 4. The logarithms of the SVs are more suitable for graphical representation; the SVs from the forward and backward EFA for the Cu(II)/ox/en system are plotted in figure 1. The number of species is determined visually, while the SVs caused by an absorbing substance show a sharp increase as a function of pH, which is due to the evolution of a species; those caused by error factor increase only very little. Figure 1 shows that six major components

*Table 3. Log(SV) of forward EFA.*

| pH | $SV_1$ | $SV_2$ | $SV_3$ | $SV_4$ | $SV_5$ | $SV_6$ | $SV_7$ | $SV_8$ |
|---|---|---|---|---|---|---|---|---|
| 4·0 | 0·5313 | 0·0064 | 0 | 0 | 0 | 0 | 0 | 0 |
| 4·5 | 0·6536 | 0·0075 | 0·0056 | 0 | 0 | 0 | 0 | 0 |
| 5·0 | 0·7401 | 0·0251 | 0·0070 | 0·0050 | 0 | 0 | 0 | 0 |
| 5·5 | 0·8157 | 0·0400 | 0·0086 | 0·0053 | 0·0017 | 0 | 0 | 0 |
| 6·0 | 0·8655 | 0·1095 | 0·0134 | 0·0053 | 0·0022 | 0·0012 | 0 | 0 |
| 6·5 | 0·8944 | 0·1925 | 0·0310 | 0·0061 | 0·0031 | 0·0022 | 0·0012 | 0 |
| 7·0 | 0·9079 | 0·2770 | 0·0585 | 0·0064 | 0·0039 | 0·0031 | 0·0020 | 0·0011 |
| 7·5 | 0·9189 | 0·3541 | 0·0700 | 0·0066 | 0·0045 | 0·0038 | 0·0029 | 0·0020 |
| 8·0 | 0·9315 | 0·4232 | 0·0755 | 0·0067 | 0·0045 | 0·0039 | 0·0035 | 0·0028 |
| 8·5 | 0·9440 | 0·4707 | 0·0783 | 0·0067 | 0·0048 | 0·0041 | 0·0036 | 0·0028 |
| 9·0 | 0·9570 | 0·5086 | 0·0800 | 0·0068 | 0·0051 | 0·0042 | 0·0038 | 0·0034 |
| 9.5 | 0·9741 | 0·5454 | 0·0811 | 0·0068 | 0·0051 | 0·0043 | 0·0038 | 0·0034 |
| 10·0 | 0·9933 | 0·5777 | 0·0821 | 0·0077 | 0·0057 | 0·0050 | 0·0043 | 0·0037 |
| 10·5 | 1·0153 | 0·6017 | 0·0832 | 0·0078 | 0·0057 | 0·0050 | 0·0043 | 0·0038 |
| 11·0 | 1·0414 | 0·6227 | 0·0842 | 0·0081 | 0·0058 | 0·0050 | 0·0044 | 0·0038 |

| pH | $SV_9$ | $SV_{10}$ | $SV_{11}$ | $SV_{12}$ | $SV_{13}$ | $SV_{14}$ | $SV_{15}$ | $SV_{16}$ |
|---|---|---|---|---|---|---|---|---|
| 4·0 | 0 | 0 | 0 | 0 | 0 | 0 | 0 | 0 |
| 4·5 | 0 | 0 | 0 | 0 | 0 | 0 | 0 | 0 |
| 5·0 | 0 | 0 | 0 | 0 | 0 | 0 | 0 | 0 |
| 5·5 | 0 | 0 | 0 | 0 | 0 | 0 | 0 | 0 |
| 6·0 | 0 | 0 | 0 | 0 | 0 | 0 | 0 | 0 |
| 6·5 | 0 | 0 | 0 | 0 | 0 | 0 | 0 | 0 |
| 7·0 | 0 | 0 | 0 | 0 | 0 | 0 | 0 | 0 |
| 7·5 | 0·0011 | 0 | 0 | 0 | 0 | 0 | 0 | 0 |
| 8·0 | 0·0020 | 0·0009 | 0 | 0 | 0 | 0 | 0 | 0 |
| 8·5 | 0·0020 | 0·0012 | 0·0009 | 0 | 0 | 0 | 0 | 0 |
| 9·0 | 0·0027 | 0·0020 | 0·0010 | 0·0007 | 0 | 0 | 0 | 0 |
| 9·5 | 0·0029 | 0·0027 | 0·0014 | 0·0010 | 0·0007 | 0 | 0 | 0 |
| 10·0 | 0·0029 | 0·0028 | 0·0015 | 0·0012 | 0·0010 | 0·0007 | 0 | 0 |
| 10·5 | 1·0030 | 0·0029 | 0·0016 | 0·0015 | 0·0011 | 0·0008 | 0·0002 | 0 |
| 11·0 | 1·0035 | 0·0030 | 0·0023 | 0·0015 | 0·0012 | 0·0011 | 0·0007 | 0 |

*Table 4. Log(SV) of backward EFA.*

| pH | $SV_1$ | $SV_2$ | $SV_3$ | $SV_4$ | $SV_5$ | $SV_6$ | $SV_7$ | $SV_8$ |
|---|---|---|---|---|---|---|---|---|
| 3·5 | 1·0414 | 0·6227 | 0·0842 | 0·0081 | 0·0058 | 0·0050 | 0·0044 | 0·0038 |
| 4·0 | 0·9910 | 0·5835 | 0·0793 | 0·0081 | 0·0055 | 0·0050 | 0·0043 | 0·0035 |
| 4·5 | 0·9530 | 0·5324 | 0·0737 | 0·0080 | 0·0052 | 0·0049 | 0·0043 | 0·0035 |
| 5·0 | 0·9163 | 0·4571 | 0·0610 | 0·0074 | 0·0051 | 0·0043 | 0·0035 | 0·0034 |
| 5·5 | 0·8889 | 0·3715 | 0·0530 | 0·0073 | 0·0051 | 0·0043 | 0·0034 | 0·0034 |
| 6·0 | 0·8645 | 0·2538 | 0·0288 | 0·0071 | 0·0043 | 0·0038 | 0·0034 | 0·0032 |
| 6·5 | 0·8360 | 0·1377 | 0·0139 | 0·0061 | 0·0043 | 0·0038 | 0·0033 | 0·0024 |
| 7·0 | 0·7998 | 0·0397 | 0·0139 | 0·0060 | 0·0042 | 0·0034 | 0·0026 | 0·0018 |
| 7·5 | 0·7580 | 0·0204 | 0·0086 | 0·0047 | 0·0038 | 0·0027 | 0·0019 | 0·0011 |
| 8·0 | 0·7102 | 0·0204 | 0·0056 | 0·0044 | 0·0028 | 0·0020 | 0·0012 | 0 |
| 8·5 | 0·6532 | 0·0182 | 0·0056 | 0·0039 | 0·0025 | 0·0016 | 0 | 0 |
| 9·0 | 0·6003 | 0·0170 | 0·0054 | 0·0039 | 0·0017 | 0 | 0 | 0 |
| 9·5 | 0·5461 | 0·0150 | 0·0052 | 0·0018 | 0 | 0 | 0 | 0 |
| 10·0 | 0·4757 | 0·0132 | 0·0024 | 0 | 0 | 0 | 0 | 0 |
| 10·5 | 0·3904 | 0·0038 | 0 | 0 | 0 | 0 | 0 | 0 |

| pH | $SV_9$ | $SV_{10}$ | $SV_{11}$ | $SV_{12}$ | $SV_{13}$ | $SV_{14}$ | $SV_{15}$ | $SV_{16}$ |
|---|---|---|---|---|---|---|---|---|
| 3·5 | 0·0035 | 0·0030 | 0·0023 | 0·0015 | 0·0012 | 0·0011 | 0·0007 | 0 |
| 4·0 | 0·0034 | 0·0029 | 0·0022 | 0·0015 | 0·0012 | 0·0008 | 0·0006 | 0 |
| 4·5 | 0·0034 | 0·0029 | 0·0022 | 0·0014 | 0·0011 | 0·0008 | 0 | 0 |
| 5·0 | 0·0030 | 0·0022 | 0·0014 | 0·0013 | 0·0008 | 0 | 0 | 0 |
| 5·5 | 0·0026 | 0·0021 | 0·0013 | 0·0008 | 0 | 0 | 0 | 0 |
| 6·0 | 0·0023 | 0·0013 | 0·0008 | 0 | 0 | 0 | 0 | 0 |
| 6·5 | 0·0018 | 0·0009 | 0 | 0 | 0 | 0 | 0 | 0 |
| 7·0 | 0·0009 | 0 | 0 | 0 | 0 | 0 | 0 | 0 |
| 7·5 | 0 | 0 | 0 | 0 | 0 | 0 | 0 | 0 |
| 8·0 | 0 | 0 | 0 | 0 | 0 | 0 | 0 | 0 |
| 8·5 | 0 | 0 | 0 | 0 | 0 | 0 | 0 | 0 |
| 9·0 | 0 | 0 | 0 | 0 | 0 | 0 | 0 | 0 |
| 9·5 | 0 | 0 | 0 | 0 | 0 | 0 | 0 | 0 |
| 10·0 | 0 | 0 | 0 | 0 | 0 | 0 | 0 | 0 |
| 10·5 | 0 | 0 | 0 | 0 | 0 | 0 | 0 | 0 |

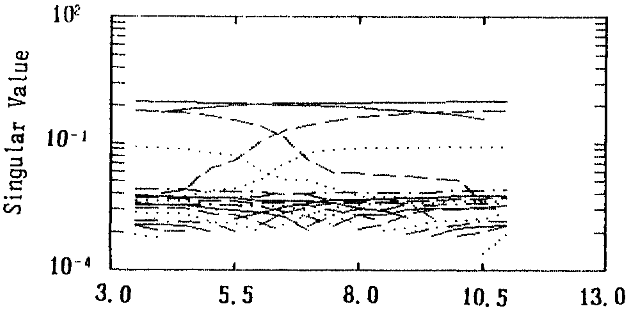

*Figure 1. Submatrix analysis of the Cu(II)/ox/en system.*

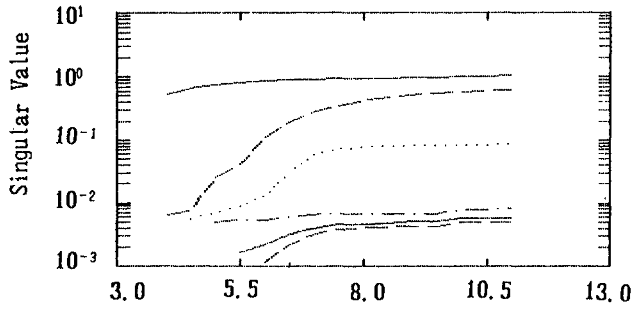

*Figure 2. Submatrix analysis of the Cu(II)/ox/en system for the six species of figure 1.*

can be distinguished from other minor components, all evolving from the error factors. The error factors are restricted to the lower part of the figure. Cutting the EFA plot at logarithmic singular values lower than $10^{-3}$ yielded clear graph (figure 2), and confirmed that there were only six significant components present. A combined plot of the forward and backward analysis was used to demonstrate where the components arise and where they disappear. Figure 2 demonstrates that new factors, 2, 3, 4, 5, 6, appear at around pHs of 4·5, 5·0, 5·5, 6·0 and 6·5. No further factors were observed until the pH was 12. The first factor can be related to free Cu(II) ions because it was present from the beginning of the titration.

Two binary subsystems were investigated by submatrix analysis in the same way. The forward and backward EFA plots showed the appearance and disappearance of a second and a third new factor at around pH 4·5 and 5·0 in the Cu(II)/ox system. These two factors can be attributed to the complex formed between Cu(II) and oxalate, [Cu(ox)] and $[Cu(ox)_2]^{2-}$. Two species had a strong influence upon factors 2 and 3 in the Cu(II)/en

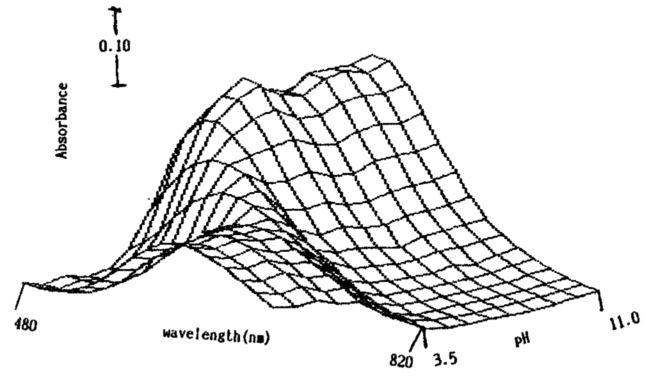

*Figure 3. Three-dimensional plot of the spectrophotometric titration data for Cu(II)/ox/en system.*

system; these were two complexes which were formed between Cu(II) and ethylendiamine, $[Cu(en)]^{2+}$ and $[Cu(en)_2]^{2+}$.

Investigation of binary subsystems and chemical reasoning may lead to an association of factors 1, 2, 3, 4, 5 and 6 with the formation of Cu(II), Cu(ox), $[Cu(ox)_2]^{2-}$, Cu(en)(ox), $[Cu(en)]^{2+}$ and $[Cu(en)_2]^{2+}$ in the Cu(II)/ox/en system. In fact, factor analysis expresses the original data matrix in terms of linear combination of orthogonal vectors. These independent vectors define a space that contains the same information as the data matrix. Associated with each eigenvector is a descriptor of its importance, which is referred to as the eigenvalue. Therefore, all absorbing species have some effect on all the eigenvalues. This was clear in figure 2 at around pH 5·0. $[(Cu(ox)_2]^{2-}$ was responsible for the introduction of factor 3 and also influenced factor 2. Similarly, at around pH 5·5, Cu(en)(ox) introduced factor 4 and influenced factors 2 and 3.

Figure 3 shows a three-dimensional plot of the experimental data matrix $D$ obtained in the system of Cu(II)/ox/en. There was no variation of the absorption band due to the Cu(II) until a pH value of around 4·5–5·0 was reached; this was indicative of the beginning of complexation. In the pH range where complexation took place, around 4·5–11, the absorption band moved towards lower wavelengths and there was an increase in intensity.

### Conclusions

Evolving factor analysis (EFA) is a powerful method using spectrophotometric titration for the study of speciation of multiple equilibria systems. The method does not require any assumptions about the chemical model of the equilibrium system. Based on mathematical algorithm of EFA, a program called SPGREFA was designed to investigate the speciation of the Cu(II)/ox/en system. Submatrix analyses are used to estimate the number of species and to show where the species arise and where they disappear. Based on the algorithm of PCA and some newer error functions recommended by Malinowski and others, a program called SPGRAFA was written to estimate the number of species in the system. The results showed that the system contained six species of the type Cu(II), [Cu(ox)], $[Cu(ox)_2]^{2-}$, [Cu(en)(ox)], $[Cu(en)]^{2+}$ and $[Cu(en)_2]^{2+}$. This paper demonstrates that the method is an effective way of studying complex systems in environmental samples.

### Acknowledgement

The authors would like to thank the National Natural Science Foundation of China for financial support.

### References

1. Legget, J. (Ed), *Computational Methods for the Determination of Formation Constants* (Plenum, New York, 1985).
2. Gampp, H., Maeder, M., Meyer, C. J. and Zuberbuhler, A. D., *Talanta*, **33** (1986), 943.
3. Malinowski, E. R. and Howery, D. E., *Factor Analysis in Chemistry* (Wiley, New York, 1985).
4. He, X., Li, H. and Shi, H., *Fenxi Huaxue*, **14** (1986), 34.
5. Malinowski, E. R., *Journal of Chemometrics*, **1** (1987), 33.
6. Gemperline, P. J. and Hamilton, J. C., In *Computer-Enhanced Analytical Spectroscopy*, Vol. 2, H. L. C. Meuzelaar, (Ed). (Plenum, New York, 1990).

